# Dominance of multidrug resistant CC271 clones in macrolide-resistant *streptococcus pneumoniae *in Arizona

**DOI:** 10.1186/1471-2180-12-12

**Published:** 2012-01-18

**Authors:** Jolene R Bowers, Elizabeth M Driebe, Jennifer L Nibecker, Bette R Wojack, Derek S Sarovich, Ada H Wong, Pius M Brzoska, Nathaniel Hubert, Andrew Knadler, Lindsey M Watson, David M Wagner, Manohar R Furtado, Michael Saubolle, David M Engelthaler, Paul S Keim

**Affiliations:** 1 Translational Genomics Research Institute, Flagstaff, AZ, USA; 2 Laboratory Sciences of Arizona, Tempe, AZ, USA; 3 Northern Arizona University, Flagstaff, AZ, USA; 4 Life Technologies, Foster City, CA, USA; 5 University of Arizona, Tucson, AZ, USA

## Abstract

**Background:**

Rates of resistance to macrolide antibiotics in *Streptococcus pneumoniae *are rising around the world due to the spread of mobile genetic elements harboring *mef*(E) and *erm*(B) genes and post-vaccine clonal expansion of strains that carry them.

**Results:**

Characterization of 592 clinical isolates collected in Arizona over a 10 year period shows 23.6% are macrolide resistant. The largest portion of the macrolide-resistant population, 52%, is dual *mef*(E)/*erm*(B)-positive. All dual-positive isolates are multidrug-resistant clonal lineages of Taiwan^19F^-14, mostly multilocus sequence type 320, carrying the recently described transposon Tn*2010*. The remainder of the macrolide resistant *S. pneumoniae *collection includes 31% *mef*(E)-positive, and 9% *erm*(B)-positive strains.

**Conclusions:**

The dual-positive, multidrug-resistant *S. pneumoniae *clones have likely expanded by switching to non-vaccine serotypes after the heptavalent pneumococcal conjugate vaccine release, and their success limits therapy options. This upsurge could have a considerable clinical impact in Arizona.

## Background

*Streptococcus pneumoniae *is a major etiological agent of pneumonia, otitis media, sinusitis, and other respiratory pathology. Macrolides remain a primary antibiotic choice for physicians treating such infections due to their broad spectrum of activity, patient tolerance, easy outpatient treatment, high achievable tissue concentrations, and anti-inflammatory properties. Use of macrolides has led to increased rates of resistance in *S. pneumoniae *[1,2] and even clinical treatment failure in several cases [3-5]. Macrolide resistance rates in clinical isolates of *S. pneumoniae *vary greatly among countries [6-9].

The main mechanisms of macrolide resistance in *S. pneumoniae *also vary geographically. The *erm*(B) encoded methylation of the ribosomal macrolide target site, which confers high-level macrolide resistance as well as resistance to lincosamides and streptogramin B (MLS_B _phenotype), is the prevalent mechanism in some Asian, European, Middle Eastern, and African countries [6,9-13]. The *mef *encoded efflux pump conferring low-level macrolide resistance (M phenotype) is more prevalent in other Asian and European countries and North America [9,14-16].

*S. pneumoniae *clones carrying both genes (dual-positive) have emerged as important clinical populations. These strains have serotypes not covered by the heptavalent pneumococcal conjugate vaccine (PCV7) released in 2000 and are multidrug resistant, posing a significant health threat. [9,10,15,17,18]. These dual-positive *S. pneumoniae *strains now comprise a substantial portion of macrolide resistant isolates in regions across the globe [6,7,9,11,19].

A primary vehicle for lateral transfer of both genes is Tn*2010*, a transposon of the tetracycline resistance gene *tet*(M)-carrying Tn*916 *family with an inserted *erm*(B) element and *mef*(E)-containing mega element [20]. A second transposon carrying both *erm*(B)and *mef*(E), Tn*2017*, comprised of Tn*916 *with the *erm*(B)-carrying Tn*917 *and the mega element inserted, was found in a Hungarian isolate from 2003 [21]. Tn*916*-family transposons with various insertions are the basis of most *erm*(B)-carrying mobile genetic elements, while *mef*(E) is known to be only in variants of the mega element [20].

In this study, we characterize a set of macrolide resistant *S. pneumoniae *clinical isolates collected in Arizona based on *mef*(E) and *erm*(B) gene presence, multilocus sequence typing (MLST) and serotyping, antibiotic susceptibility profiles, and potential transposon carriage. We document likely episodes of capsule switching and serotype replacement, both mechanisms that allow *S. pneumoniae *to evade the PCV7 and cause infection in an immunized population.

## Methods

### Bacterial isolates

From 1999 to 2008, 592 *S. pneumoniae *isolates were collected by a large hospital reference laboratory that receives specimens from ten system-wide medical centers and a high volume private reference laboratory that receives specimens from regional inpatient, long-term care, and outpatient facilities. Isolates considered non-invasive were obtained from upper respiratory tract (upper respiratory specimens plus sinus, nasal, and nasopharyngeal swabs), lower respiratory tract, ear, eye, body fluid, wound, and tissue (n = 488). Isolates considered invasive were obtained from blood (n = 100), urine (n = 2), and cerebrospinal fluid (CSF, n = 2) specimens. All were identified by bile solubility and optochin susceptibility testing. Patients ranged in age from 1 month to 88 years with a median age of 19 years and mean age of 29 1/2years.

### Antimicrobial susceptibility testing

In vitro susceptibility testing followed Clinical and Laboratory Standards Institute (CLSI) recommended methodologies and interpretational zone of inhibition diameter and minimal inhibitory concentration (MIC) breakpoints [22]. Susceptibilities were determined for most isolates for penicillin, erythromycin, clindamycin, tetracycline, and trimethoprim-sulfamethoxazole by disk agar-diffusion (Kirby-Bauer), manual microdilution (MicroScan, Siemens Healthcare Diagnostics, Inc., Deerfield, IL), or gradient strip agar diffusion (E-test, AB Biodisk, Stockholm, Sweden) testing.

### DNA extraction

Bacterial DNA was extracted for PCR using DNeasy Blood and Tissue Kit (Qiagen, Valencia, CA) following manufacturer's instructions for Gram-positive bacteria with the addition of 200U of mutanolysin (Sigma-Aldrich, St. Louis, MO).

### Real-time PCR

Isolates were screened with commercial real-time PCR assays to detect *mef*(E), *mef*(A), *erm*(B), and *tet*(M) (Life Technologies, Foster City, CA). Real-time PCR was carried out in 10 μL reactions containing 5 μL 2X Taqman Universal PCR Mastermix (Life Technologies, Foster City, CA), 0.5 μL 20X assay mix, and 0.2 ng genomic DNA template. Screening was done on the 7900HT (Life Technologies, Foster City, CA) using the following thermal cycling conditions: 50°C for 2 min, 95°C for 10 min, and 40 cycles of 95°C for 15 s, 60°C for 1 min.

### Multilocus sequence typing and serotyping

Multilocus sequence typing (MLST) was performed using primer pairs described in the MLST database http://spneumoniae.mlst.net/[23]. Allele profiles and sequence types were also obtained from the database. Strains differing by one of the seven MLST loci were designated single-locus variants (SLVs).

PCR deduction of serotypes was performed on select isolates as described at http://www.cdc.gov/ncidod/biotech/strep/pcr.htm[24-27], with the addition of a previously described PCR to differentiate serotype 6A from 6B [28].

### Transposon detection PCR

Primers previously described, some with slight modifications to adjust melting temperatures, were used to detect regions of transposons known to carry antibiotic resistance genes (Table 1). In brief, selected isolates were subject to PCR using primers for the genes *int *and *xis*, and *tnpR *and *tnpA *to detect the presence of transposons in the Tn*916 *and Tn*917 *families respectively [29]. Depending on their resistance gene profile, some isolates positive for only *Tn916 *were subject to PCR using the following primer pairs: SG1 and LTf [30] to substantiate the presence of Tn*2009 *or Tn*2010 *with a 1 kb PCR product, EB2 [31] and TN2 [32] to confirm Tn*2010 *with a 3.3 kb PCR product, and J12 and J11 to detect and differentiate Tn*6002 *(3.6 kb PCR product) from Tn*6003/*Tn*1545 *(7.9 kb PCR product) [33]. Isolates positive for both transposon families were subject to PCR using primers J12 and J11 to detect Tn*3872 *with an 800 bp PCR product. Amplicon presence or absence and sizes analyzed via gel electrophoresis guided the identification of transposon presence and type; authors concede these are presumptions based on published transposon maps and therefore limited data.

**Table 1:** Oligonucleotides used in transposon detection

Transposon region	Oligo nucleotide	Sequence	Amplicon size (bp)	Transposon presumed present	*S. pneumoniae *population screened	Reference
*int *gene	int_for	GCGTGATTGTATCTCACT	1046	Tn*916*	Dual-positive, *erm*(B)-positive, *mef*(E)-positive	[29]

	int_rev	GACGCTCCTGTTGCTTCT				[29]

*xis *gene	xis_for	AAGCAGACTGAGATTCCTA	193	Tn*916*	Dual-positive, *erm*(B)-positive, *mef*(E)-positive	[29]

	xis rev	GCGTCCAATGTATCTATAA				[29]

*tnp*Rgene	O21	CCAAGGAGCTAAAGAGGTCCC	1528	Tn*917*	Dual-positive, *erm*(B)-positive, *mef*(E)-positive	[29]

	O22	GTCCCGAGTCCCATGGAAGC				[29]

*tnp*A gene	O23	GCTTCCATGGGACTCGGGAC	2115	Tn*917*	Dual-positive, *erm*(B)-positive, *mef*(E)-positive	[29]

	O24	GCTCCCAATTAATAGGAGA				[29]

Spans insert of *erm*(B) elements in Tn*916*	J12^d^	ATTCCCATTGAAGACGCAGAAGT	800	Tn*3872*	*erm*(B)-positive that are Tn*916*-positive	[34]

	J11^d^	CTACCGCACTTCGTTTGGTGTAC	3600	Tn*6002*		[34]

			7900	Tn*6003 *or Tn*1545*		

Junction of mega insert and Tn*916*	SG1	CTCACTGCACCAGAGGTGTA	1000	Tn*2009 *or Tn*2010*	Dual-positive and *mef*(E)-positivie that are Tn*916*-positive	[30]

	LTf	GCAGAGTATACCATTCACATCGAAGTTCCAC				30]

Junction of *erm*(B) element and Tn*916*	EB2	AGTAATGGTACTTAAATTGTTTAC	3300	Tn*2010*	Dual-positive that are Tn*916*-positive	[31]

	TN2^a^	GAAGTA(G/C)AAGCTAAAGATGG				[32]

^a ^Modified from original to change melt temperature or incorporate degeneracies

## Results

### Macrolide resistance

In our collection of 592 *S. pneumoniae *isolates, 140 (23.6%) are erythromycin resistant, including only 5 of the 104 invasive isolates. Within the erythromycin resistant population, at least 110 (78.6%) are multidrug resistant, defined here as resistant to antibiotics in at least 3 different classes or 2 classes and positive for the *tet*(M) gene if not tested for tetracycline susceptibility.

Of the 140 erythromycin resistant strains, 44 (31.4%) were *mef*(E)-positive including three invasive isolates, 13 (9.3%) were *erm*(B)-positive including one invasive isolate, and 73 (52.1%) were dual *mef*(E)/*erm*(B)-positive including one invasive isolate. One isolate was positive for *mef*(A). Nine (6.4%) were negative for the macrolide resistance genes and no further analyses were conducted to determine their resistance mechanisms. Thirty-eight of the *mef*(E)-positive isolates expressed the M-phenotype while six expressed the MLS_B _phenotype, manifesting alternative clindamycin resistance mechanisms. All 13 *erm*(B)-positive isolates showed MLS_B_. Sixty-eight of the dual-positive isolates showed MLS_B_; the remaining five expressed the M-phenotype suggesting clindamycin resistance is inducible or *erm*(B) is non-functional in these isolates.

Ten of the 452 erythromycin susceptible isolates were *mef*(E)-positive, one was *erm*(B)-positive, and five were dual-positive, signifying a loss of gene function in these isolates.

### Time series

Macrolide resistance rates in our collection increased from 1999 to 2004, then stabilized through 2008 (Table 2).

**Table 2:** Time series of macrolide resistance gene presence, sequence types, and serotypes in *S. pneumoniae *clinical isolates

						Time period			
	**1999-2000**		**2001-2002**	**2003-2004**		**2005-2006**		**2007-2008**	

**Macrolide-resistant *S. pneumoniae *population**	**Sequence types (no. isolates)**	**Serotyp e^a ^(no. tested)**	**No. isolates**	**Sequence types (no. isolates)**	**Serotype^a ^(no. tested)**	**Sequence types (no. isolates)**	**Serotype^a ^(no. tested)**	**Sequence types (no. isolates)**	**Serotype^a ^(no. tested)**

Dual *mef*(E)/*erm*(B)-positive	271 (4)	19 F (4)	0	320^b ^(2)	**19A **(2)	320^b ^(19)	**19A **(16)	320^b ^(24)	**19A **(21)
	
	1412^b,e ^(1)	19 F (1)		1396^b ^(2)	19 F (2)	320^b,e ^(2)	**19A **(1)	1459^b ^(1)	NT
	
	3039^b,g ^(1)	19 F (1)		271 (1)	19 F (1)	271 (2)	19 F (2)	NF^b ^(1)	**19A **(1)
	
	NF^b,c ^(2)	19 F (2)		NT (1)	19 F (1)	1459^b ^(2)	19 F (1)	NT^e ^(1)	NT
	
	NT^d ^(1)	19 F (1)				3039^b ^(1)	19 F (1)		
	
						1396^b,e ^(1)	19 F (1)		
	
						NF^b ^(3)	19 F (2)		
	
						NT (1)			

Total for time period	9 (39.1%)			6 (40.0%)		31 (58.5%)		27 (67.5%)	

*mef*(E)-positive	236^b ^(2)	19 F (2)	0	376 (2)	**6A **(2)	2705 (3)	**33A/F/37 **(3)	3280 (3)	NT
	
	13^g ^(1)	NT		1186 (2)	NT	1186 (3)	NT	1379 (2)	**6C **(2)
	
	156 (1)	**6A **(1)		1556 (1)	NT	236^b ^(2)	19 F (2)	162^f ^(1)	NT
	
	376 (1)	**6A **(1)		6422 (1)	NT	156 (1)	9 V (1)	199 (1)	**19A **(1)
	
	384^f ^(1)	6B (1)		NT^f ^(1)	**6C**	199 (1)	**19A **(1)	344 (1)	NT
	
	384^g ^(1)	6B (1)				558 (1)	**35B **(1)	1518 (1)	6B (1)
	
	NF^g ^(1)	NT				1379 (1)	**6C **(1)	NF (1)	**6A **(1)
	
	NT (2)	NT				3065 (1)	**6C **(1)		
	
	NT^f ^(1)	NT				NF^f ^(1)	19 F (1)		
	
						NT (1)	**6C **(1)		
	
						NT^f ^(1)	NT		

Total for time period	11 (47.8%)			7 (46.7%)		16 (30.2%)		10 (25.0%)	

*erm*(B)-positive	315 (2)	6B (2)	0	63 (1)	**15A/15 F **(1)	63 (5)	**15A/15 F **(5)	63 (2)	**15A/15 F **(2)
	
	3066^g ^(1)	18A/B/C/F (1)		NT (1)		180 (1)	**3 **(1)		

Total for time period	3 (13.1%)			2 (13.3%)		6 (11.3%)		2 (5.0%)	

*mef*(A)-positive								1111 (1)	**6C **(1)

Total for time	0		0	0		0		1 (2.5%)	

Total macrolide resistant/Total no. isolates collected	23/131 (17.6%)	0/34 (0%)	15/54 (27.8%)	53/223 (23.8%)	40/150 (26.7%)				

^a ^Serotype deduced by PCR; serotypes in bold are non-vaccine types

^b ^Sequence type is a single locus variant of ST271

^c ^NF, Sequence type not found in MLST database

^d ^NT, Not typed

^e ^Dual-positive with M-phenotype (n = 5)

^f ^*mef*(E)-positive with MLS_B _phenotype (n = 6)

^g ^Invasive isolate (n = 5)

Dual-positive numbers grew steadily over the 10-year duration of the study from 39.1% to 67.5% of all macrolide resistant isolates. Concurrently, the proportion *mef*(E)-positive fell (47.8% to 25.0%) and the proportion of *erm*(B)-positive remained relatively steady until 2007-2008 (Table 2).

**Figure 1:**
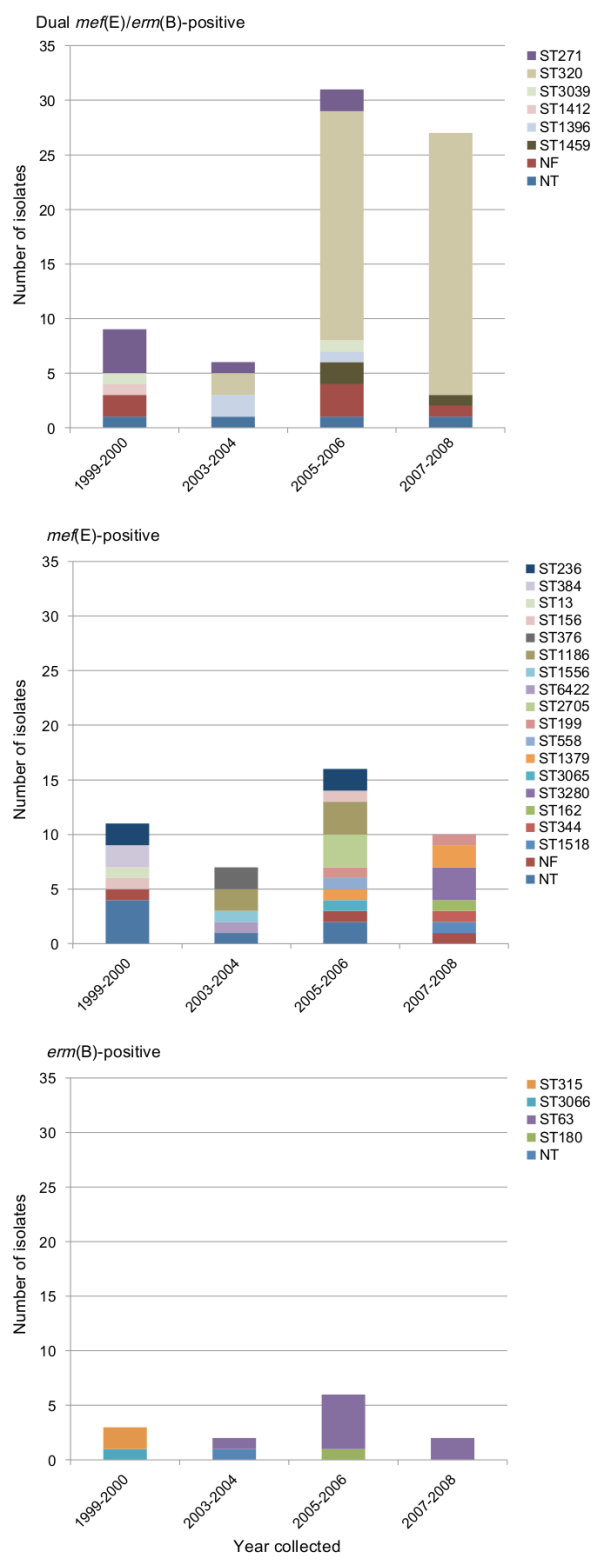
**Changes in population structure over time in dual *mef*(E)/*erm*(B)-positive, *mef*(E)-positive, and *erm*(B)-positive *S. pneumoniae *clinical isolates**. No isolates positive for *mef*(E) or *erm*(B) genes were collected in 2001-2002. ST, sequence type; NF, sequence type not found; NT, not typed

According to MLST and serotype deduction, strain dominance and diversity changed for all three populations over the 10 years (Table 2, Figure 1). The most prevalent sequence types of the early dual-positive population include ST271 and various single locus variants (SLVs) that all belong to clonal complex (CC) 271. Through 2004, all isolates (n = 15) in this population except the two ST320 isolates serotyped as 19 F, a vaccine type (VT). ST320, a SLV of ST271, became dominant in our collection more recently, and almost exclusively by 2008. Of the 39 ST320 isolates serotyped, all were found to be a non-vaccine type (NVT) serotype 19A. This is consistent with the well-documented serotype switch in *S. pneumoniae *isolates in the U.S. [35,36].

Sequence types and serotypes of the *mef*(E)-positive population remained diverse over the time period (Table 2, Figure 1). Out of 20 total sequence types identified in this population, only six were found in more than one two-year period, three of those in both pre- and post-vaccine introduction time periods. These include ST236, serotype 19 F, the genotype of the highly dispersed Taiwan^19F^-14 clone and likely ancestor to the CC271 lineages, ST376 of NVT 6A, and ST156, the genotype of the Spain^9V^-3 clone in which serotype switching from VT 9 V to NVT 19A has been documented [35]. Interestingly, in the pre-vaccination time period, the ST156 strain is serotype 6A while the strain from the post-vaccination time period likely is 9 V. (PCR deduction typed the strain as 9 V or 9 F.) The former was isolated from a 70 year-old male who may have received the 23-valent polysaccharide pneumococcal vaccine (PPSV) intended for adults over 65 years old and high-risk groups, and which covers serotype 9 V. This strain may have switched from 9 V to 6A in response to PPSV, before introduction of PCV7. Additionally, the *mef*(E)-positive population illustrates serotype replacement. Historically VT strains caused most pneumococcal disease, however after 2000, more NVT strains than VT strains were found.

In the *erm*(B)-positive population, serotype replacement may also be evident. The early population is comprised of two ST315, VT 6B strains and a ST3066 strain, possibly VT 18 C. (This isolate typed as 18A, B, C, or F using PCR; the Pneumococcal Molecular Epidemiology Network [PMEN] clone database links ST3066 with serotype 18 C [37].) They were replaced in later years by the unrelated ST63, NVT 15A or 15 F (PMEN links ST63 with serotype 15A [37]) and ST180, NVT 3 (Table 2, Figure 1).

### *mef*(E) and *erm*(B) population characteristics: Specimen types

Many (n = 32) of the dual *mef*(E)/*erm*(B)-positive isolates were from ear specimens collected after 2000 (post-PCV7) from children of vaccine age (less than five years old after the introduction of the PCV7 in 2000). Many (n = 32) were from respiratory specimens, only eight of which came from children of vaccine age; most came from adults post-PCV7. Similarly, a relatively large proportion of isolates of the *erm*(B)-positive population was from ear (n = 4) or eye (n = 3) specimens from children of vaccine age, while most of the rest are from adult patients. In contrast, the *mef*(E)-positive population consists mostly of respiratory isolates (n = 25), and a large fraction of these (n = 21) are from older generations. A relatively small proportion was from ear specimens (n = 4) or eye specimens (n = 6) of children of vaccine age.

### mef(E) and erm(B) population characteristics: MLST, antimicrobial resistance, transposon carriage

Analyses of genotypes of dual-positive isolates showed little diversity within the population; seven of the 73 dual *mef*(E)/*erm*(B)-positive isolates are ST271 and the remaining are SLVs of ST271 (Table 2). All 73 of the dual-positive isolates are multidrug-resistant. Most are resistant to penicillin, erythromycin, clindamycin, and trimethoprim-sulfamethoxazole and positive for *tet*(M); five were reported clindamycin-susceptible. Thirty-three dual-positive isolates representing all sequence types found were analyzed for transposon carriage. All 33 were positive for the genes *int *and *xis*, and with primer sets SG1/LTf and EB2/TN2, and negative for the genes *tnpR *and *tnpA*, indicating carriage of Tn*2010*, the transposon known to harbor *erm*(B), *mef*(E), and *tet*(M) (Table 3). None apparently carried Tn*2017*. Although the dual positive population is the largest, it exhibits the lowest diversity of genotypes.

**Table 3:** Mobile genetic elements carrying *erm*(B) and *mef*(E) genes and associated genotypic and phenotypic profiles

Population Dual *mef*(E)/*erm*(B)-	Sequence type 320	Antibiotic susceptiblity profile^a ^Pen^ns^Ery^r^Cli^r^Tet^r/u+^Sxt^r^	Presumed transposon (no. isolates with same profile tested) Tn*2010 *(12)	Total no. isolates of same profile 43
	320	Pen^ns^Ery^r^Cli^s^Tet^r/u+^Sxt^r^	Tn*2010 *(1)	2
	
	320	Pen^ns^Ery^r^Cli^r^Tet^u-^Sxt^r^	Tn*2010 *(1)	2
	
	271	Pen^ns^Ery^r^Cli^r^Tet^r/u+^Sxt^r^	Tn*2010 *(5)	7
	
	1396	Pen^r^Ery^r^Cli^r^Tet^ns/u+^Sxt^r^	Tn*2010 *(2)	2
	
	1396	Pen^r^Ery^r^Cli^s^Tet^ns/u+^Sxt^r^	Tn*2010 *(1)	1
	
	1459	Pen Ery Cli Tet Sxt	Tn*2010 *(3)	3
	
	3039	Pen^r/u^Ery^r^Cli^r^Tet^r/u+^Sxt^r^	Tn*2010 *(2)	2
	
	1412	Pen^r^Ery^r^Cli^s^Tet^r^Sxt^r^	Tn*2010 *(1)	1
	
	NF	Pen^ns^Ery^r^Cli^r^Tet^r/u+^Sxt^r^	Tn*2010 *(5)	6
	
	NT	Pen^ns^Ery^r^Cli^r^Tet^r/u+^Sxt^r^	NT	3

*mef*(E)-positive	NT	Pen^ns^Ery^r^Cli^s^Tet^r^Sxt^r^	NT	1

	236	Pen^ns^Ery^r^Cli^s^Tet^r/u+^Sxt^r^	Tn*2009 *(4)	4

	1186	Pen^s^Ery^ns^Cli^s^Tet^s/u-^Sxt^r^	mega (4)	4

	1186	Pen^ns^Ery^r^Cli^s^Tet^u-^Sxt^r^	mega (1)	1

	1379	Pen^ns^Ery^r^Cli^s^Tet^s^Sxt^r^	mega (2)	2

	1379	Pen^s^Ery^r^Cli^s^Tet^s^Sxt^r^	mega (1)	1

	2705	Pen^s^Ery^r^Cli^s^Tet^s/u-^Sxt^r^	mega (3)	3

	3280	Pen^ns^Ery^r^Cli^s^Tet^r/u+^Sxt^r^	Tn*2009 *(3)	3

	376	Pen^ns^Ery^r^Cli^s^Tet^s/u-^Sxt^r^	mega (3)	3

	156	Pen^ns^Ery^r^Cli^s^Tet^s/u-^Sxt^r^	mega (2)	2

	199	Pen^s^Ery^r^Cli^s^Tet^s^Sxt^s^	mega (1)	1

	199	Pen^ns^Ery^r^Cli^s^Tet^u-^Sxt^r^	mega (1)	1

	384	Pen^r^Ery^r^Cli^r^Tet^s+^Sxt^r^	Tn*916*+mega (1)	1

	384	Pen^r^Ery^r^Cli^s^Tet^r^Sxt^s^	Tn*916*+mega (1)	1

	13	Pen^r^Ery^r^Cli^s^Tet^s^Sxt^r^	mega (1)	1

	162	Pen^ns^Ery^r^Cli^r^Tet^u-^Sxt^s^	mega (1)	1

*erm*(B)-positive	344	Pen^ns^Ery^r^Cli^s^Tet^u+^Sxt^r^	Tn*916*+mega (1)	1

	558	Pen^ns^Ery^r^Cli^s^Tet^s^Sxt^s^	mega (1)	1

	1518	Pen^ns^Ery^r^Cli^s^Tet^s^Sxt^r^	mega (1)	1

	1556	Pen^r^Ery^r^Cli^s^Tet^u-^Sxt^r^	mega (1)	1

	3065	Pen^s^Ery^r^Cli^s^Tet^u-^Sxt^ns^	mega (1)	1

	6422	Pen^r^Ery^r^Cli^s^Tet^r^Sxt^r^	mega (1)	1

	NF	Pen^r^Ery^r^Cli^s^Tet^s^Sxt^ns^	mega (1)	1

	NF	Pen^ns^Ery^r^Cli^s^Tet^s^Sxt^s^	mega (1)	1

	NF	Pen^ns^Ery^r^Cli^ns^Tet^u+^Sxt^r^	Tn*2009 *(1)	1

	NT	Pen^ns^Ery^r^Cli^r^Tet^u+^Sxt^s^	Tn*916*+mega (1)	1

	NT	Pen^s^Ery^r^Cli^s^Tet^s^Sxt^r^	mega (1)	1

	NT	Pen^s^Ery^r^Cli^s^Tet^s^Sxt^s^	mega (2)	2

	NT	Pen^r^Ery^r^Cli^r^Tet^r^Sxt^r^	Tn*916*+mega (1)	1

	NT	Pen^r^Ery^r^Cli^r^Tet^u-^Sxt^r^	mega (1)	1

	63	Pen^ns/u^Ery^r^Cli^r^Tet^r/u+^Sxt^s/u^	*erm*(B) element (8)	8

	315	Pen^i^Ery^r^Cli^r^Tet^r^Sxt^i^	Tn*916 *family (2)	2

	3066	Pen^s^Ery^r^Cli^r^Tet^r^Sxt^s^	Tn*3872 *(1)	1

	180	Pen^s^Ery^r^Cli^r^Tet^r^Sxt^s^	Tn*916 *family (1)	1

	NT	Pen^s^Ery^r^Cli^r^Tet^r^Sxt^s^	Tn*3872 *(1)	1

^a ^Pen, penicillin; Ery, erythromycin; Cli, clindamycin; Tet, tetracycline; Sxt, trimethoprim-sulfamethoxazole; r, resistant; ns, nonsusceptible; s, susceptible; u, unknown phenotype; +, positive for *tet*(M) by PCR; -, negative for *tet*(M) by PCR; NF, not found; NT, not typed

The *erm*(B)-positive population is comprised of strains of four distinct sequence types, none of which match any from the dual-positive population (Table 2). Only one MLST allele is common to both populations. Despite MLST dissimilarity among the *erm*(B)-positive isolates, all have similar antibiotic susceptibility profiles. Most are intermediately or fully susceptible to penicillin and trimethoprim-sulfamethoxazole while resistant to erythromycin and clindamycin, and all carry *tet*(M). Out of the 13 isolates in this population, all eight ST63 isolates were negative for *int, xis, tnpR*, and *tnpA*; the genetic context of their antibiotic resistance genes remains unknown. Two isolates, one ST3066 and a non-typed isolate, tested positive for Tn*916 *and Tn*917*, and produced an 800 bp PCR product with J12/J11 primers, signifying the presence of Tn*3872*. The two ST315 isolates and the ST180 isolate tested positive for Tn*916*, but were negative for Tn*917 *and with J12/J11, possibly indicating carriage of *tet*(M) in Tn*916 *and a separate *erm*(B) element (Table 3).

Genotype analyses of the *mef*(E)-positive population show high diversity with relatively even distribution. Besides three sets of SLVs, the highest number of MLST alleles shared by any two sequence types is three, and no more than four isolates of the same sequence type were identified. Many different antibiotic susceptibility profiles were identified in this population, with no single dominant profile. Of the 44 *mef*(E)-positive isolates, eight isolates of three sequence types, ST236, a SLV of ST236, and ST3280, were positive for *int *and *xis*, for the SG1/LTf region, and for *tet*(M), indicating the presence of Tn*2009*. Five others were positive for only *int *and *xis *and *tet*(M), indicating carriage of Tn*916 *and a separate mega element. The absence of these transposon PCR targets and *tet*(M) in the other 31 isolates suggests they are carrying the mega element (Table 3).

## Discussion

Macrolide resistance rates in clinical isolates of *S. pneumoniae *vary greatly among countries. The rate in our collection of isolates from Arizona patients, 23.6%, is consistent with other studies targeting *S. pneumoniae *in North America [15,38].

The temporal trend in *mef*(E) and *erm*(B) prevalence that we observed in our collection, the rise in proportion dual gene-positive inversely to the proportion *mef*(E)-positive, is similar to those of other non-invasive isolate studies [39]. Recent studies of invasive isolates have shown low rates of dual gene carriage and multidrug resistance [11,14,40]. Likewise, only one of the invasive isolates we tested was dual-gene positive. These significant differences between invasive and non-invasive isolate gene carriage and susceptibility profiles may arise because macrolide-induced selection pressures on invasive *S. pneumoniae *may be different from those on non-invasive *S. pneumoniae*, due to the pharmacodynamics of macrolide antibiotics.

Over half of our macrolide resistant *S. pneumoniae *isolates are positive for both *erm*(B) and *mef*(E). All these dual-positive strains belong to CC271, have almost identical multidrug resistance profiles, and are likely carrying Tn*2010*. Clonal lineages of multidrug-resistant *S. pneumoniae *belonging to CC271 are now distributed worldwide and make up a significant portion of the macrolide resistant *S. pneumoniae *isolates in many regions [7,10,14,41,42]. The emergence of these clones is at least partly a response to introduction of PCV7, in which lineages of the successful multidrug resistant Taiwan^19F^-14 ST236 clone acquired *erm*(B) and switched serotypes in response to the selective pressures of an immunized population [6,43]. One cosmopolitan lineage recombined into ST320 and serotype 19A [35,36]. This clone has afflicted Arizona children since the PCV7 release in 2000; of the 73 dual-positive isolates in our collection, 47 are ST320, 38 of which are from children of vaccine age. Most of these are from ear and respiratory specimens, an observation consistent with that of the global PROTEKT studies [6,15]. These data display the opportunistic dominance of a few *S. pneumoniae *clones in the post-PCV7 era. The pervasiveness of the multidrug resistant phenotype poses a serious public health concern for increased treatment failure and selection of these clones with the usage of any one of several antibiotics.

Genotyping our collection revealed high strain diversity within the *mef*(E)-positive population. The variety of antibiotic susceptibility profiles and mobile genetic elements carrying *mef*(E) reflect the sequence type and serotype diversity found in this population. These data indicate that *mef*(E)-carrying *S. pneumoniae *are the ancestral macrolide-resistant strains in the U.S. Serotype replacement and a possible serotype switching event are evident in this population; NVTs outnumber VTs in later time periods, and ST156, the identifier of the Spain^9V^-3 clone, typed as NVT 6A. One notable observation of the *mef*(E)-positive population is that the latest ST236 seen is 2005-2006, more evidence that this clone acquired the *erm*(B) gene, and its lineages now comprise the dual *mef*(E)/*erm*(B)-positive population.

Genotype analyses of the small *erm*(B)-positive population illustrate serotype replacement. ST315, VT 6B is not seen after 2000, while ST63, NVT 15A became dominant [37]. These findings could be the result of loss in ST315 or acquisition in ST63 of *erm*(B) and consequent sampling bias, however neither strain carries *erm*(B) in a Tn*917*-family transposon leaving the mobility of the *erm*(B) element in these strains unknown.

The dramatic increase in *erm*(B)-carrying *S. pneumoniae *isolates is important in regions where *mef*-carrying isolates have historically predominated. Treatment with macrolides is an option for patients suffering localized infections caused by *mef*-carrying *S. pneumoniae*, as drug concentrations in tissues can supercede these bacteria's macrolide MICs [44,45]. However, macrolide MICs for *erm*(B)-carrying strains are significantly higher than those of *mef*-carrying isolates [46], increasing the need for alternative antibiotics where *erm*(B) predominates. It remains to be seen whether the U.S. will see an increase in clinical failure in macrolide-treated cases parallel to the increase in *erm*(B)-carrying *S. pneumoniae*.

## Conclusions

Our Arizona-based study supports other global studies that illustrate the impact that PCV7 has had on the population structure of macrolide resistant *S. pneumoniae *in non-invasive isolates, and calls attention to the longevity of the success of particular multidrug resistant clones. The vaccine has reduced morbidity and mortality and multidrug resistance in invasive disease, but serotype replacement and serotype switching by *S. pneumoniae *has eclipsed these effects in non-invasive disease, and may soon for invasive disease [8,35,47,48]. However, the recently released PCV13, which covers serotypes of the newly dominant multidrug-resistant clones, including 19A, may have very different consequences for *S. pneumoniae *population genetics. Vaccine response and population genetics studies are important to our understanding of *S. pneumoniae *evolution and strain dominance. More accessible higher resolution technology, for example whole genome sequencing, provides us with more information than MLST, resistance gene profiling, targeted transposon investigation, and serotyping combined [49]. Consequently, future studies that include next generation sequencing would help to better and more quickly elucidate the effects of *S. pneumoniae *infection prevention and treatment strategies.

## Authors' contributions

JRB participated in the molecular data collection, analysis, and interpretation, and drafted the manuscript. EMD designed the study and was involved in critically revising the manuscript. JLN participated in the molecular data collection and analysis. BRW conducted the microbiological methods and analyzed and interpreted data. DSS participated in data collection and was involved in critically revising the manuscript. AHW and PMB designed the assays and methods for real-time PCR. NH and AK participated in molecular data collection, analysis and interpretation. LMW participated in data collection and analysis. DMW participated in data collection and was involved in critically revising the manuscript. MRF, MS, DME, and PSK conceived of and designed the study. All authors read and approved the final manuscript.
